# Domiciliary Cockroaches as Carriers of Human Intestinal Parasites in Lagos Metropolis, Southwest Nigeria: Implications for Public Health

**Published:** 2018-06-13

**Authors:** Adedotun A Adenusi, Mary I Akinyemi, Dele Akinsanya

**Affiliations:** 1 Medical Parasitology Unit, Department of Medical Microbiology and Parasitology, College of Medicine, University of Lagos, Idi-Araba, Surulere, Lagos, Nigeria; 2 Statistics Unit, Department of Mathematics, University of Lagos, Akoka, Lagos, Nigeria; 3 Department of Zoology, University of Lagos, Akoka, Lagos, Nigeria

**Keywords:** Cockroaches, *Periplaneta americana*, *Blattella germanica*, Intestinal parasites, Lagos

## Abstract

**Background:**

Domiciliary cockroaches are obnoxious pests of significant medical importance. We investigated the prevalence of human intestinal parasites in cockroaches and its attendant public health importance.

**Methods:**

Overall, 749 cockroaches (*Periplaneta americana*, 509, *Blattella germanica*, 240) caught by trapping from 120 households comprising 3 different housing types in Somolu, Lagos metropolis, southwest Nigeria, in 2015 were screened for human intestinal parasites using standard parasitological techniques.

**Results:**

The prevalence of human intestinal parasites in cockroaches was 96.4%. There was no statistically significant difference (P> 0.05) in parasite prevalences between *P. americana* (95.7%) and *B. germanica* (97.9%). Parasite species identified and their prevalence were as follows: *Entamoeba histolytica/dispar* (44.1%), *E. coli* (37.8%), *Giardia lamblia* (18.7%), *Cryptosporidium* sp. (13.8%), *Ascaris lumbricoides* (61.3%), *Trichuris trichiura* (55.8%), hookworms (11.6%), *Strongyloides stercoralis* (11.7%), *Taenia/Echinococcus* spp. (10.5%), *Enterobius vermicularis* (17.2%) and *Hymenolepis nana* (11.6%). Parasite prevalence and burdens varied with housing type; the prevalence was significantly higher statistically (P< 0.05) in cockroaches from low-cost bungalow, LCB (100%) and low-cost, 2-storey, LC2-S (100%) houses than in medium-cost flats, MCF (81.3%). Parasite burdens were also significantly higher statistically (P< 0.05) in cockroaches from LCB or LC2-S than in cockroaches from MCF. Parasite prevalences between cockroach gut and body surfaces were not statistically significant (P> 0.05) but mean parasite burdens in gut were significantly higher statistically (P< 0.05) than on body surfaces.

**Conclusion:**

Cockroaches types carry transmissive stages of human intestinal parasites and may act as reservoirs and potential mechanical vectors for disease transmission.

## Introduction

Cockroaches (Insecta: Blattaria) are insects, which have been in existence since antiquity (1), thriving in so many habitats and consuming virtually any organic matter, including fresh and processed human foods, stored products, garbage, and sewage (1, 2). About 4600 described species of cockroaches are distributed worldwide (3). However, only a few of the about 30 synanthropic species are considered as pests in homes, grocery stores, hospitals, offices, schools, warehouses and other establishments (4). The American cockroach, *Periplaneta americana* (Blattaria: Blattidae) and the German cockroach, *Blattella germanica* (Blattaria: Blattellidae) are considered two of the most common and notorious cosmopolitan pest species in Nigeria (5) and globally (6–8).

Cockroaches are pests of significant medical, veterinary and public health importance. Their presence and sight may induce psychological stress, the levels of which tend to be proportional to cockroach size and number (2). They are an important source of potent environmental aeroallergens, which provoke allergic reactions and exacerbate acute asthma, especially in predisposed atopic individuals (9, 10). Cockroaches contaminate foods to which they have access with their feces and foul-smelling secretions, thereby making them offensive and unsafe for human consumption (1, 2). They also serve as intermediate hosts to a number of helminth parasites of veterinary importance, some of which cause debilitating diseases in domestic animals (2).

Although cockroaches have yet, to be incriminated as biological vectors of human pathogens, their biology - filthy habits, indiscriminate diet, feeding mechanisms and morphology, make them vulnerable and suitable, at least, to acquire, mechanically transport and disseminate pathogens. Indeed, a variety of pathogenic and potentially pathogenic bacteria, fungi, and parasites have been isolated from body surfaces and/or gut of cockroaches in domestic, food-handling, and hospital environments (6, 11–14). The potential exists, therefore, for mechanical transmission through physical dislodgement, regurgitation, or fecal pellet deposition onto and/or into exposed human food, which may be, ready-to-eat or improperly cooked. Although the direct involvement of cockroaches in the transmission of parasites to humans remains to be fully established, their importance in parasite transport and dissemination cannot be underestimated.

Intestinal parasitoses are among the most common and widespread diseases of humans globally responsible for considerable morbidity and mortality, especially in children, the most vulnerable population (15). They remain a serious threat to public health worldwide, particularly in communities in resource-poor developing countries in the tropics and subtropics where high prevalences are attributable to poverty, poor living conditions, lack of potable water supply, inadequate waste disposal, poor sanitation and environmental hygiene (16). While a few of these parasites require intermediate hosts, many are transmitted by direct ingestion of infective cysts, and oocysts (protozoa) or eggs and/or larvae (helminths) in foods (especially fruits and vegetables), water, soil, pica, or on hands so contaminated.

The incidence of human intestinal parasitoses has continued to increase in recent years, in spite of concerted efforts at reduction. Moreover, the transmissive/human-infective stages of some of these parasites can survive in the environment for considerable lengths of time. Because cockroaches carry the same pathogens found in substrates with which they have contact (17), it is plausible that cockroaches, in environments contaminated with parasite cysts, oocysts, eggs and or larvae, may pick up these stages for transport and dissemination.

Poor household hygiene and inadequate environmental sanitation provide congenial atmosphere for cockroach infestation. Somolu, Lagos metropolis, southwest Nigeria, typifies a cosmopolitan setting in a developing economy such as Nigeria, where poor sanitary conditions, together with ecology and demography, provide congenial atmosphere for cockroach infestation and contact with pathogens. In spite of the above, and the heterogeneity of Somolu in terms of human population and physical infrastructures, and considering the medical and public health importance of cockroaches, there is yet, no information on prevalence of human intestinal parasites in domiciliary cockroaches from this locality.

The objective of this study was to determine and compare prevalences and species composition of human intestinal parasites in cockroaches from different residential buildings in Somolu, Lagos State, southwest Nigeria.

## Materials and Methods

### Study Area

The study was carried out between Aug and Nov 2015 in Somolu (geographical coordinates, 6°32’ N and longitude 3°22’ E), a densely populated cosmopolitan area of Lagos metropolis, and headquarters of Somolu Local Government Area (LGA) of Lagos State, southwest Nigeria. The LGA which has a land size of 11615km^2^ and a population of 403569 (18) is inhabited predominantly by people of the Yoruba extraction, although all tribes and sub-tribes of the Nigerian nationality and expatriates are also resident.

Climate in the area is typical of that in the State and is characterized generally by daily temperatures of 24–34 °C and monthly relative humidity of 64–93% during the rainy season, usually from Apr to Oct, and 34–49% during the dry season from Nov to Mar.

### Housing Types

Houses in Somolu are typical, a mix of old and modern architecture. One hundred and twenty residential buildings were selected for the study using a stratified random sampling procedure. They comprised 40 each, of buildings classified as low-cost bungalow (LCB), low-cost, 2-storey (LC2-S), and medium-cost flat (MCF), based on building architecture and socio-economic status of residents. Each LCB or LC2-S had between 5 and 7 rooms on either side of a story and each room was occupied by low-income class individual(s)/family who shared the only kitchen and toilet/bathroom facilities on either side of a story. Each MCF consists of a 3-bedroom apartment with a kitchen and at least one toilet facility, in a semi-detached building inhabited by a medium-income class family. Inclusion criterion was that no insecticide and/or trapping device was used to treat cockroach infestation in the one week prior to the commencement of the study. Advocacy visits were made to residents of selected houses to explain the objective(s) of the study and to seek for their participation, cooperation, and understanding in the execution of the study.

### Cockroach collection and identification

Cockroaches were trapped live, using sterile jars baited with pieces of bread soaked in a small amount of beer. The jars, whose inside upper portions were coated with a thin film of petroleum jelly (Vaseline^®^) to prevent cockroach escape, were placed indoors at 19:00h and retrieved at 07:00h the next morning, for 1–3 consecutive days. Cockroaches were transported in the jars to the laboratory where they were anaesthetized and killed by exposure to chloroform fume. They were examined under a dissecting microscope and identified to the lowest taxon possible, using standard taxonomic keys (19). They cockroaches were counted and sorted by capture site (housing type), and the appropriate taxon.

### Isolation of parasites from cockroach body surface

In order to dislodge parasite stages (cysts, oocysts, eggs and/or larvae) from body surfaces, cockroaches were washed individually by submersion in 5–10ml sterile physiological saline and vortexing at low speed for 2 min. Cockroaches were removed from wash solutions using sterile forceps, fixed in 70% alcohol for 5min and air-dried at room temperature. Wash solutions were centrifuged at 2000g for 5min, supernatants were decanted and the bottom 0.5–1ml processed further using the formol-ether concentration technique (20). The resulting sediment mixed with the bottom 0.5ml was placed on slide, stained with Lugol’s iodine and examined microscopically for human intestinal parasite stages.

For the demonstration of *Cryptosporidium* oocysts, a modified Ziehl-Neelsen staining method (21) was used. Briefly, air-dried smears prepared from processed body surface washings were fixed with methanol and stained with carbol-fuchsin for 30min. Smears were washed with tap water, decolorized with 1% acid alcohol for 1min, washed again with tap water and counter-stained with 1% methylene blue for 1min. Smears were rinsed finally in tap water and air-dried.

Cockroach specimens which could not be processed immediately were kept in the freezer at −4 °C.

### Isolation of parasites from cockroach gut

Following washing in physiological saline, fixing in 70% alcohol and subsequent air-drying, each cockroach was placed in a sterile Petri dish and dissected under a dissecting microscope using sterile entomological needles. Whole gut was removed and homogenized in 2–5ml physiological saline. The homogenate was filtered through gauze and centrifuged at 2000g for 5min, following which the supernatant was decanted. The bottom 0.5–1ml was processed further using the formol-ether concentration technique. The resulting sediment mixed with the bottom 0.5ml was placed on slide, stained with Lugol’s iodine and examined microscopically as described above.

*Cryptosporidium* oocysts were identified following the modified Ziehl-Neelsen staining method described above.

### Parasite Identification

Cysts, oocysts, eggs and/or larvae of human intestinal parasites were identified microscopically using bench aids (22) and their numbers recorded. A cockroach was considered a carrier if any parasite stage was detected in preparations from body surface and/or gut contents.

### Data analysis

Data were input into Microsoft Excel and analyzed using the “R: A Language and Environment for Statistical Computing” software package (23). Analysis of variance (ANOVA) was used to test for differences in overall prevalence of parasites between cockroach species, in overall burdens of parasites on cockroach body surfaces and in gut, and in overall burdens of protozoan and helminth groups of parasites. The Tukey’s HSD test was used for multiple comparisons of overall prevalences or burdens of protozoan and/or helminth parasites between pairs of residential building types. All tests were carried out at 95% significance level; in all cases, a P< 0.05 was considered statistically significant.

## Results

### Prevalence of human intestinal parasites by cockroach species

Overall, 749 cockroaches comprising two species, *P. americana* (509) and *B. germanica* (240) were caught and identified. Human intestinal parasite stages were identified in 96.4% of the cockroaches (Table 1). There was no statistically significant difference (F-statistic= 2.354, P= 0.125) in overall prevalence of parasites between *P. americana* (95.7%) and *B. germanica* (97.9%). Parasites were more frequently isolated from cockroaches trapped from LCB and LC2-S households than in cockroaches from MCF (81.3%) (Table 1). There was no statistically significant difference (P= 1.00) in prevalence of parasites between cockroaches from LCB (100%) and LC2-S (100%) households while there were statistically significant differences in prevalences of parasites between cockroaches from LCB and MCF (P=0.001) and LC2-S and MCF (P=0.001).

**Table 1.** Prevalence of human intestinal parasite stages in cockroaches

**Housing Type**	**Number of cockroaches examined (% parasite +ve)**		**Total**

	***Periplaneta americana***	***Blattella germanica***	
**LCB**	207 (100)	91 (100)	298 (100)^a^
**LC2-S**	202 (100)	105 (100)	307 (100)^a^
**MCF**	100 (78.0)	44 (88.6)	144 (81.3)^b^
**Total**	509 (95.7)^a^	240 (97.9)^a^	749 (96.4)

* Values with same superscript along the same column or row are not significantly different statistically at α= 0.05

### Species diversity of human intestinal parasites in cockroaches

**Fig. 1.**
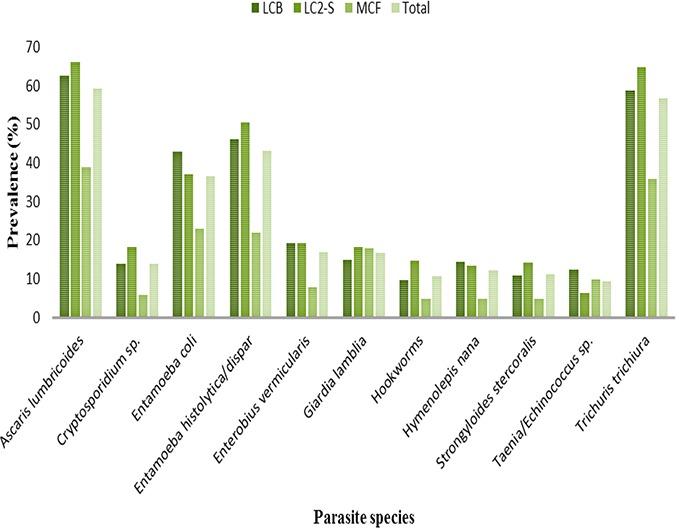
Prevalence of human intestinal parasites in *Periplaneta americana* from different housing types

**Fig. 2.**
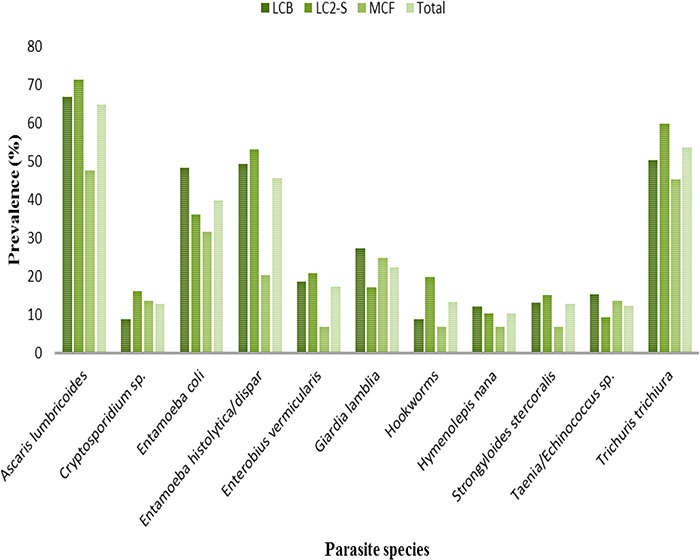
Prevalence of human intestinal parasites in *Blattella germanica* from different housing type

Eleven human intestinal parasites, comprising four protozoan and seven helminth species were identified on body surfaces and/or in gut of cockroaches. The species and their respective prevalences in both *P. americana* and *B. germanica* are as follows: *E. histolytica/dispar* (44.1%), *E. coli* (37.8%), *G. lamblia* (18.7%), *Cryptosporidium* sp. (13.8%), *A. lumbricoides* (61.3%), *T. trichiura* (55.8%), hookworms (11.6%), *S. stercoralis* (11.7%), *Taenia/Echinococcus* spp. (10.5%), *E. vermicularis* (17.2%) and *H. nana* (11.6%). Prevalences of these parasite species in *P. americana* and *B. germanica*, trapped from different housing types are shown in Figs. 1 and 2, respectively. The helminths, *A. lumbricoides* and *T. trichiura*, and the protozoans, *E. histolytica/dispar* and *E. coli* were the four most prevalent species in both species of cockroaches, across all housing types, save *B. germanica* from MCF wherein *G. lamblia* was the fourth most prevalent species (25.0%). Their respective overall prevalences in *P. americana* were 59.5%, 56.8%, 43.2% and 36.7% (Fig. 1) while the corresponding overall prevalences in *B. germanica* were 65.0%, 53.8%, 45.8% and 40.0% (Fig. 2).

### Prevalence and burden of human intestinal parasites on cockroach body surfaces and in the gut

Parasite prevalence in cockroach gut was either a little higher than or identical numerically, to the corresponding prevalence on body surface. The ANOVA test showed no statistically significant differences between prevalences in gut and on body surfaces in *P. americana* (F= 1.363, P= 0.243), *B. germanica* (F= 0.344, P= 0.558) or both (F= 1.671, P= 0.196).

Overall parasite prevalences were significantly higher statistically with helminths than protozoans in *P. americana* (93.9% *vs*. 83.7%, F= 28.25, P= 1.31e-07), *B. germanica* (97.5% *vs*. 83.3%, F= 29.38, P= 9.46e-08), or both (95.1% *vs*. 83.6%, F= 54.52, P= 2.54e-13) and in cockroaches from LCB (P= 0.005), LC2-S (P= 3.45e-07) and MCF (P= 0.0001).

Mean parasite burdens/counts in *P. americana* (Table 2) and *B. germanica* (Table 3) varied with cockroach body region, parasite group (protozoa or helminth) and housing type. Highest parasite burdens on cockroach body surfaces and in gut were with the protozoans, *E. histolytica/dispar* and *E. coli* and the helminths, *A. lumbricoides* and *T. trichiura*, overall and in each housing type (Tables 2, 3).

**Table 2.** Mean parasite burdens in *Periplaneta americana* cockroaches from houses in Somolu, Lagos, Nigeria

**Parasite species**	**LCB (N=207)**			**LC2-S (N=202)**			**MCF (N=100)**		
		
	**Body surface**	**Gut**	**Total**	**Body surface**	**Gut**	**Total**	**Body surface**	**Gut**	**Total**
***Ascaris lumbricoides***	2.2±2.1	4.8±4.2	7.0±6.2	2.3±2.0	4.9±4.2	7.2±6.2	0.9±1.2	1.5±2.0	2.3±3.2
***Cryptosporidium* sp**	0.3±0.8	0.6±1.6	0.9±2.3	0.5±1.1	0.9±2.1	1.3±3.1	0.1±0.3	0.2±0.6	0.2±1.0
***Entamoeba coli***	1.3±1.7	2.9±3.7	4.2±5.4	1.1±1.6	2.3±3.4	3.3±4.9	0.4±0.9	0.9±1.7	1.3±2.6
***Entamoeba histolytica/dispar***	1.4±1.9	3.1±3.8	4.5±5.6	1.7±1.9	3.3±3.8	5.0±5.6	0.5±1.0	0.9±1.8	1.3±2.8
***Enterobius vermicularis***	0.5±1.1	1.0±2.2	1.5±3.3	0.5±1.2	1.0±2.1	1.5±3.3	0.2±0.6	0.3±1.0	0.4±1.6
***Giardia lamblia***	0.3±0.9	0.7±1.7	1.0±2.6	0.5±1.0	0.9±2.1	1.4±3.1	0.3±0.6	0.5±1.2	0.8±1.8
**Hookworms**	0.2±0.6	0.4±1.3	0.6±1.9	0.4±1.0	0.7±1.8	1.0±2.7	0.1±0.4	0.2±0.8	0.2±1.1
***Hymenolepis nana***	0.3±0.8	0.6±1.5	0.8±2.3	0.3±0.8	0.5±1.5	0.8±2.3	0.1±0.3	0.1±0.6	0.2±0.9
***Strongyloides stercoralis***	0.2±0.5	0.3±1.0	0.5±1.5	0.2±0.6	0.5±1.2	0.7±1.8	0.0±0.2	0.1±0.6	0.2±0.8
***Taenia/Echinococcus* sp**	0.3±0.8	0.6±1.7	0.8±2.4	0.2±0.7	0.5±1.5	0.5±2.1	0.1±0.5	0.3±1.0	0.4±1.5
***Trichuris trichiura***	1.9±1.9	3.9±3.8	5.8±5.7	2.2±2.0	4.4±3.8	6.7±5.8	0.7±1.2	1.3±2.0	2.1±3.1
**Total**	8.8±4.1	18.8±7.5	27.6±11.4	9.7±3.8	19.7±7.0	29.3±10.5	3.3±2.6	6.2±4.6	9.4±7.1

**Table 3.** Mean parasite burdens in *Blattella germanica* cockroaches from houses in Somolu, Lagos, Nigeria

**Parasite species**	**LCB (N=91)**			**LC2-S (N=105)**			**MCF (N=44)**		
		
	**Body surface**	**Gut**	**Total**	**Body surface**	**Gut**	**Total**	**Body surface**	**Gut**	**Total**
***Ascaris lumbricoides***	2.7±2.2	5.7±4.7	8.4±6.9	2.6±2.0	5.6±4.1	8.3±6.0	1.2±1.5	2.1±2.5	3.3±4.0
***Cryptosporidium* sp**	0.2±0.6	0.3±1.1	0.5±1.7	0.4±1.0	0.8±2.1	1.2±3.0	0.2±0.5	0.3±0.9	0.5±1.4
***Entamoeba coli***	1.6±1.9	3.4±4.0	4.9±5.8	1.0±1.6	2.1±3.2	3.1±4.7	0.8±1.2	1.2±2.0	2.0±3.2
***Entamoeba histolytica/dispar***	1.6±1.8	3.4±4.0	5.0±5.8	1.7±1.8	3.4±3.7	5.0±5.4	0.5±1.0	0.7±1.5	1.2±2.5
***Enterobius vermicularis***	0.5±1.0	1.0±2.3	1.5±3.3	0.6±1.3	1.1±2.3	1.8±3.5	0.1±0.5	0.2±0.8	0.3±1.2
***Giardia lamblia***	0.7±1.3	1.4±2.7	2.2±4.0	0.4±1.1	0.9±2.4	1.4±3.5	0.5±0.9	0.8±1.4	1.2±2.2
**Hookworms**	0.3±0.9	0.5±1.9	0.8±2.8	0.4±1.0	0.9±2.0	1.3±3.0	0.1±0.5	0.2±0.8	0.3±1.2
***Hymenolepis nana***	0.2±0.7	0.5±1.4	0.7±2.2	0.3±0.9	0.5±1.6	0.8±2.4	0.1±0.3	0.2±0.8	0.3±1.1
***Strongyloides stercoralis***	0.2±0.6	0.4±1.1	0.6±1.6	0.3±0.7	0.5±1.3	0.8±1.9	0.1±0.4	0.3±1.0	0.4±1.5
***Taenia/Echinococcus* sp**	0.3±0.8	0.7±1.8	1.0±2.6	0.1±0.4	0.2±1.0	0.3±1.4	0.2±0.5	0.4±1.2	0.6±1.6
***Trichuris trichiura***	1.6±1.9	3.5±3.8	5.1±5.6	2.0±2.0	4.2±3.9	6.3±5.9	0.9±1.2	1.6±2.0	2.5±3.1
**Total**	9.8±4.8	20.9±9.7	30.7±14.3	9.9±3.2	20.3±6.4	30.2±9.4	4.4±2.6	8.0±4.1	12.4±6.6

Overall mean parasite burdens in gut were significantly higher statistically than on body surfaces in *P. americana* (F= 330, P< 2e-16), *B. germanica* (F= 166.3, P< 2e-16), and both (F= 496, P< 2e-16). Similarly, overall burdens of helminth parasites were significantly higher statistically than those of protozoan parasites in *P. americana* (F= 156, P< 2e-16), *B. germanica* (F =74.89, P< 2e-16), both (F= 229.8, P< 2e-16) and in LCB (P= 0.0001), LC2-S (P= 0.0001) and MCF (P= 0.044). Differences in overall parasite burdens were statistically significant between LCB and MCF (P= 0.0001) and LC2-S and MCF (P= 0.0001), but not statistically significant between LCB and LC2-S (P= 0.996).

## Discussion

Cockroaches are nuisance pests whose activities impact negatively on humans. Of great concern to human and public health, is their capability as potential mechanical vectors of pathogens, including parasites. Previous studies from other parts of Nigeria (13, 24, 25) and elsewhere (6, 12, 26) had reported that cockroaches captured from homes, hostels, hospitals, and markets carry an array of human intestinal parasites. Results of the present study, which show clearly that the two cockroach species (*P. americana* and *B. germanica*) from residential buildings in Somolu, Lagos, southwest Nigeria, carry human intestinal parasites on their body surfaces and/or in the gut indicate that concerns over their potential and/or role as mechanical vectors cannot be overlooked.

The species of human intestinal parasites recovered from cockroaches in the present study, (*E. histolytica/dispar*, *E. coli*, *G. lamblia*, *Cryptosporidium* sp. *A. lumbricoides*, *T. trichiura*, hookworms, *S. stercoralis*, *Taenia/Echinococcus* spp., *H. nana*, and *E. vermicularis*) are responsible for a number of disease conditions in man, some of which could be life-threatening. The three major soil-transmitted helminths (*A. lumbricoides*, *T. trichiura*, and hookworms) account for a high burden of disease globally and are intimately related with malnutrition, growth stunting and cognitive deficits in children (27). *Strongyloides stercoralis* may cause complicated infections with high case fatality rates due to hyper-infection or dissemination, especially in immunocompromised individuals (28). *Cryptosporidium* sp. and *G. lamblia* are nowadays, major causes of diarrhoea, especially in children (29). *Entamoeba histolytica* causes amoebiasis, a potentially severe and life-threatening disease and the second most common cause of death from parasitic diseases, after malaria (30). Accidental ingestion of *Taenia* (particularly, *T. solium*) eggs often results in human neurocysticercosis, the leading cause of preventable epilepsy worldwide and also, a leading cause of deaths from food-borne diseases (31). Species of *Echinococcus* cause life-threatening chronic diseases with poor prognosis and high fatality rates, if not carefully managed clinically (32).

Parasite species reported in the present study are consistent with those documented in similar studies (13, 24, 25, 33, 34). However, the disparities could be due to differences in the levels of household and environmental hygiene, transmission dynamics between study localities, and in the diagnostic procedure employed. Predominance of *A. lumbricoides* on body surfaces and/or in the gut of cockroaches across all different housing types in the present study is in consonance with findings from similar studies in other parts of the country (13, 25, 33). This could be due to its predominance in the human population and/or the persistence of its eggs in the environment for months to years (35).

Identity of parasite species in cockroaches from all three housing types indicates that these parasites have equal chances of being acquired by cockroaches, probably because they are endemic in the study area. Differences in individual parasite species burdens between cockroaches from low-cost (LCB or LC2-S) and MCF could then be explicated by the varying levels of hygiene and sanitation in each housing type. Poorer housing and sanitary conditions, which could predispose to higher parasite contamination, characterize low-cost households.

Overwhelming and widespread prevalence (96.4%) of human intestinal parasites in domiciliary cockroaches in the present study is singular in Nigeria and is a cause for public health concern. Other studies from Nigeria had reported prevalences of 58.6% in Calabar, Southsouth (25), 67.1% in Owerri, Southeast (33) and 77.5% in Sokoto, Northwest (24). Al-Mayali and Al-Yaqoobi (36) and El-Sherbini and El-Sherbini (34) reported prevalences of 83.3% and 98% respectively, in Iraq and Egypt. Disparities in prevalences between different studies may be explained by differences in the levels of hygiene and sanitation between study localities.

Identity in the diversity of parasites between *P. americana* and *B. germanica* as well as the insignificant statistical differences in their respective prevalences in the cockroach species indicate a uniform distribution of parasite species between the two cockroach species in the same environment. They also suggest that the two cockroach species have equal potential for mechanical transport and possibly, consequent dissemination of parasites in the environment.

Because pathogens carried by cockroaches are acquired from their immediate environments (37), the human intestinal parasites reported herein, were acquired through contact with unhygienic environments. Most of the parasites whose cysts, oocysts, eggs and/or larvae (hookworm: *A. duodenale* only) were isolated from cockroaches in the present study are transmitted to humans via consumption of food and/or water so contaminated. Since cockroaches travel indiscriminately between filth and human food, they may be capable of disseminating parasite stages (on their body surfaces and/or in the gut) through physical dislodgement, vomitus and/or feces onto any substrate in the environment, including human food and food preparation surfaces. The medical and public health implications of this are better imagined.

Cysts of *E. histolytica* and *E. dispar* are morphologically indistinguishable microscopically, so also are the eggs of *Echinococcus* and *Taenia* species. Since molecular techniques, which differentiate reliably, cysts of *E. histolytica* from *E. dispar* (38) and eggs of *Echinococcus* from *Taenia* species (39, 40) were not employed in the present study, the former was simply identified morphologically as *E. histolytica/dispar* and the latter as *Taenia/Echinococcus* spp.

## Conclusion

Cockroaches (*P. americana* and *B. germanica*) across different housing types in Somolu, Lagos metropolis, Nigeria, transport on their body surfaces and/or in the gut, transmissive stages of human intestinal parasites and thus, may serve as reservoirs and potential mechanical vectors for disease transmission. The exceptionally high prevalence of parasites in cockroaches (96.4%) justifies the need for improvements in existing standards of household hygiene and environmental sanitation in order to minimize cockroach contact with unhygienic sites/substrates from which parasites are acquired.
